# Supplementary material for: Development and Psychometric Evaluation of the End-of-Life Nursing Competency Scale for Clinical Nurses
Source: Healthcare (Basel). 2024 Aug 8;12(16):1580. doi: 10.3390/healthcare12161580 (PMC11354138; doi:10.3390/healthcare12161580)
Supplement: Supplementary file 1 [file healthcare-12-01580-s001.zip › healthcare-3094475-supplementary.pdf]

healthcare MDPI

Table S1. Final Tool

	Items	Strongly Disagree	Disagree	Neutral	Agree	Strongly Agree
1	I can determine the end-of-life stage according to the patient's changing condition.	1	2	3	4	5
2	I can assess a patient's changing condition by end-of-life stages.	1	2	3	4	5
3	I can provide nursing care according to the post-mortem management process.	1	2	3	4	5
4	I am familiar with the clinical signs and symptoms that indicate imminent death.	1	2	3	4	5
5	I can provide physical care according to the end-of-life stages (e.g., respiration, nutrition, excretion).	1	2	3	4	5
6	I can explain how to utilize end-of-life services within the facility.	1	2	3	4	5
7	I understand the legal and administrative processes that nurses should follow during the end-of-life process.	1	2	3	4	5
8	I understand the processes and methods related to advance directives.	1	2	3	4	5
9	I am familiar with the end-of-life nursing protocols used in the institution where I work.	1	2	3	4	5
10	I can accommodate and support the spiritual care needs of patients facing imminent death and their families.	1	2	3	4	5
11	I can communicate with patients facing imminent death and their families using facial expressions, intonation, and tone that empathizes with their emotions.	1	2	3	4	5
12	I can provide counseling to patients and families during the end-of-life stages.	1	2	3	4	5
13	I can accommodate and support the psychological and emotional reactions of patients facing imminent death and their families.	1	2	3	4	5
14	I can handle unexpected death calmly and without emotional fluctuation.	1	2	3	4	5
15	I can control my negative emotions (such as sadness, fear, avoidance, etc.).	1	2	3	4	5
16	I do not have any reluctance in caring for patients facing imminent death.	1	2	3	4	5
17	I can report cases where biomedical ethics concerning end-of-life are violated.	1	2	3	4	5
18	I can provide end-of-life nursing care while adhering to biomedical ethics.	1	2	3	4	5

Table S2. End-of-Life Nursing Competency Items with Supporting References

Items
1. I can judge the end-of-life stage according to the patient's changing condition.<br><br>Montagnini, M.; Smith, H.M.; Price, D.M.; Strodtman, L.; Ghosh, B. An instrument to assess self-perceived competencies in end-of-life care for health care professionals: the end-of-life care questionnaire. *American Journal of Hospice and Palliative Medicine*® 2021, *38*, 1426-1432.
2. I can assess the patient's changing condition according to the end-of-life stage.

Montagnini, M.; Smith, H.M.; Price, D.M.; Strodtman, L.; Ghosh, B. An instrument to assess self-perceived competencies in end-of-life care for health care professionals: the end-of-life care questionnaire. *American Journal of Hospice and Palliative Medicine*® 2021, *38*, 1426-1432.
3. I can perform nursing care according to the post-mortem management process.<br>Wilson, J.; Laverty, D.; Mann, T.; Hayes, A.R., J. Guidance for staff responsible for care after death. 2015.
4. I know the clinical symptoms and signs that appear at the imminent end of life.<br>Pfister, D.; Müller, M.; Müller, S.; Kern, M.; Rolke, R.; Radbruch, L. Validation of the Bonn test for knowledge in palliative care (BPW). *Der Schmerz* 2011, *25*, 643-653.
5. I can provide physical care according to the end-of-life stage (e.g., breathing, nutrition, excretion).<br>Norlander, L. *To comfort always: A nurse's guide to end-of-life care*, 2nd ed.; Sigma Theta Tau Intl: Indianapolis, 2015.
6. I can explain how to use end-of-life related services within the facility.<br>Norlander, L. *To comfort always: A nurse's guide to end-of-life care*, 2nd ed.; Sigma Theta Tau Intl: Indianapolis, 2015.
7. I know the legal and administrative procedures that nurses must perform at the end of life.<br>ANA. Nurses' roles and responsibilities in providing care and support at the end of life. Available online: https://www.nursingworld.org/~4af078/globalassets/docs/ana/ethics/endoflife-positionstatement.pdf (accessed on May 21).
8. I know the procedures and handling methods according to the Advance Directives for Life-Sustaining Treatment.<br>Park, E.; Kim, N. The influence of nursing professionalism, attitudes toward advance directive, and death anxiety on terminal care performance of nurses in long-term care hospitals. *Korean Journal of Adult Nursing* 2018, *30*, 183-193.
9. I know the end-of-life care protocol of the institution where I work.<br>ANA. Nurses' roles and responsibilities in providing care and support at the end of life. Available online:

https://www.nursingworld.org/~4af078/globalassets/docs/ana/ethics/endoflife-positionstatement.pdf (accessed on May 21).
10. I can accept and support the spiritual care needs of end-of-life patients and their families.<br><br>Fernández-Ortega, P.; Kav, S.; Arimón-Pagès, E. Nurses providing emotional support and spiritual care to patients and families, Spain & Turkey. *Global Perspectives in Cancer Care: Religion, Spirituality, and Cultural Diversity in Health and Healing* 2022, 86.
11. I can communicate with facial expressions, intonation, and tone that empathize with the emotions (despair, fear, sadness, etc.) of end-of-life patients and their families.<br><br>Andersson, E.; Salickiene, Z.; Rosengren, K. To be involved—A qualitative study of nurses' experiences of caring for dying patients. *Nurse Education Today* 2016, *38*, 144-149.
12. I can counsel patients and their families during the end-of-life stage.<br><br>Norlander, L. *To comfort always: A nurse's guide to end-of-life care*, 2nd ed.; Sigma Theta Tau Intl: Indianapolis, 2015.
13. I can accept and support the psychological/mental reactions of patients and their families at the end of life.<br><br>Norlander, L. *To comfort always: A nurse's guide to end-of-life care*, 2nd ed.; Sigma Theta Tau Intl: Indianapolis, 2015.
14. I can cope with unexpected end-of-life situations without emotional turmoil.<br><br>Jeong, Y.-H.; June, K.J. End of life care competencies and terminal care stress of nurses in long term care hospitals. *The Korean journal of hospice and palliative care* 2019, *22*, 125-133.
15. I can control my negative emotions (sadness, fear, avoidance, etc.).<br><br>Barr, P. Relationships of nursing stress and trait emotional intelligence with mental health in neonatal intensive care unit nurses: A cross-sectional correlational study. *Australian Critical Care* 2024, *37*, 258-264.
16. I am not averse to caring for patients facing the end of life.<br><br>Blaževičienė, A.; Laurs, L.; Newland, J.A. Attitudes of registered nurses about the end–of–life care in multi-profile hospitals: A cross sectional survey. *BMC palliative care* 2020, *19*, 1-8.

17. I can report cases where the medical ethics that must be observed during end-of-life care are violated.	Izumi, S.; Nagae, H.; Sakurai, C.; Imamura, E. Defining end-of-life care from perspectives of nursing ethics. *Nursing ethics* 2012, *19*, 608-618.
18. I provide end-of-life care in compliance with medical ethics.	Izumi, S.; Nagae, H.; Sakurai, C.; Imamura, E. Defining end-of-life care from perspectives of nursing ethics. *Nursing ethics* 2012, *19*, 608-618.
